# Impact of Nutrition on Cerebral Circulation and Cognition in the Metabolic Syndrome

**DOI:** 10.3390/nu7115477

**Published:** 2015-11-13

**Authors:** Laura Mellendijk, Maximilian Wiesmann, Amanda J. Kiliaan

**Affiliations:** 1 Department of Anatomy, Radboud University Medical Center, Donders Institute for Brain, Cognition and Behaviour, Nijmegen 6500 HB, The Netherlands; mellendijklaura@gmail.com; 2 Department of Anatomy & Geriatric Medicine, Radboud University Medical Center, Donders Institute for Brain, Cognition and Behaviour, Nijmegen 6500 HB, The Netherlands; Maximilian.Wiesmann@radboudumc.nl

**Keywords:** Metabolic Syndrome, nutrition, obesity, dyslipidemia, hypertension, hyperglycemia, cerebral circulation, cognition, dementia

## Abstract

The increasing prevalence of Metabolic Syndrome (MetS), defined as the clustering of abdominal obesity, dyslipidemia, hypertension, and hyperglycemia, appears to be driving the global epidemics cardiovascular disease (CVD) and type 2 diabetes mellitus (T2DM). Nutrition has a major impact on MetS and plays an important role in the prevention, development, and treatment of its features. Structural and functional alterations in the vasculature, associated with MetS, might form the link between MetS and the increased risk of developing CVD and T2DM. Not only does the peripheral vasculature seem to be affected, but the syndrome has a profound impact on the cerebral circulation and thence brain structure as well. Furthermore, strong associations are shown with stroke, cognitive impairment, and dementia. In this review the impact of nutrition on the individual components of MetS, the effects of MetS on peripheral and cerebral vasculature, and its consequences for brain structure and function will be discussed.

## 1. Introduction

Metabolic Syndrome (MetS) is characterized by the co-occurrence of several metabolic risk factors for cardiovascular disease (CVD), stroke, and/or type 2 diabetes mellitus (T2DM) [1,2]. In 2006, the International Diabetes Federation (IDF) estimated that 20% to 25% of the global adult population suffered from MetS and its prevalence will further increase in the coming years, driving the twin global epidemics for CVD and T2DM [2].

The relationship between insulin sensitivity, obesity, and glucose homeostasis has been observed as early as in the 1920s. At that time, a syndrome comprising hyperglycemia, hypertension, and hyperuricemia was described with insulin resistance (IR) as a possible causative factor [3]. It is almost two decades since Reaven [4] proposed that several risk factors for atherosclerotic CVD (ACVD), such as hypertension, dyslipidemia, and hyperglycemia, tend to cluster in a group he called Syndrome X. Subsequently, this syndrome was also called the insulin-resistance syndrome, since IR appeared to be central to MetS and its specific components [5]. Nowadays, a widely used name for the clustering of several metabolic components is MetS. These components comprise atherogenic dyslipidemia, hypertension, hyperglycemia, excess of central adipose tissue, and also associate with hyperuricemia, proinflammatory, and prothrombotic states. They are important markers for subsequent development of CVD and T2DM [5,6]. However, the interactions causing this clustering of metabolic risk factors are not fully understood. Associated factors are physical inactivity, atherogenic diets, advancing age, and various hormones [5]. Some investigators think that predominantly abdominal obesity and primary IR, are possibly the underlying risk factors for MetS [5,7]. Several definitions for MetS have been developed, whereof the one of the National Cholesterol Education Program Adult Treatment Panel III (NCEP/ATP III) is the most widely used, although the derived and modified International Diabetes Federation (IDF) definition is also often used. According to NCEP/ATP III, individuals are considered as having MetS when they meet at least three of the following criteria: (1) waist circumference, as measure of abdominal obesity, in men ≥102 cm (IDF 94 cm) and ≥88 cm (IDF 80 cm) in women; (2) triglyceride concentration ≥1.70 mmol/L (≥150 mg/dL) or on drug treatment for elevated triglyceride levels; (3) high-density lipoprotein cholesterol (HDL-C) <1.03 mmol/L (<40 mg/dL) in men and <1.29 mmol/L (<50 mg/dL) in women or on drug treatment for low HDL-C; (4) blood pressure ≥130/85 mmHg or on antihypertensive medication; and (5) fasting plasma glucose ≥5.6 mmol/L (≥100 mg/dL) or on drug treatment for hyperglycemia [8,9].

Individuals suffering from MetS have at least a two-fold increased risk for CVD [10] and a 1.5-fold higher risk for stroke in comparison with those without MetS [11]. The underlying mechanisms that link the components of MetS to an increased risk for CVD are not completely understood, although intermediaries have been proposed including oxidative stress, inflammation [12], arterial stiffness [13], endothelial dysfunction and increased intima media thickness (IMT) [14]. Furthermore, cerebral blood flow (CBF) seems closely related to each of MetS components; an inverse association between CBF and these cardiometabolic risk indices is found [15]. The large cerebral arteries carry a significant portion of the cerebrovascular resistance (CVR), thereby contributing to the regulation of the CBF and determining local microvascular pressure [16]. Microcirculatory changes that are more closely related to metabolic factors such as hyperglycemia, influence both the structure and function of larger vessels, potentially causing alterations in CBF [15]. Furthermore, the endothelial cells of the cerebral blood vessel walls interact intimately with neurons, astrocytes, pericytes, and glial cells forming the neurovascular unit. MetS seems to impair the cerebral vasculature via multiple interrelated mechanisms, including through the formation of atherosclerotic plaques and inflammatory processes, which eventually may lead to the disturbance of the neurovascular unit.

Several factors of MetS have also been linked to cognitive impairment and dementia [17]. Obesity for instance, is recognized as a modifiable risk factor for dementia [18,19] and also hypertension, especially mid-life hypertension, and both diabetes and prediabetes, increase the risk of developing dementia [20,21]. Possible explanations for these correlations might be found in an impaired vasculature and/or altered CBF due to MetS, which in turn leads to neuronal damage [22]. The blood-brain-barrier (BBB) forms the interface between the periphery and the brain and it is responsible for the cross-talk between both compartments. The BBB is composed of specialized endothelial cells that are reinforced by pericytes, astrocyte end feet, and extracellular matrix, which together control the transport across the BBB. Metabolic imbalance is a risk predictor associated with the decline of BBB integrity and function [23]. For example, an altered release of protein signals and factors, known as adipokines from the peripheral white adipose tissue (WAT), may impair vascular health and as well directly affect brain functioning [19].

The primary strategy for management of MetS in order to reduce the risk of developing CVD and T2DM, is consuming an overall healthy diet for weight reduction and continuous weight-maintenance [24]. Dietary patterns close to the Mediterranean diet, rich in fruits and vegetables and high in mono- and polyunsaturated fatty acids from olive oil, fish, and nuts are negatively associated with features of MetS [25,26,27,28]. General dietary recommendations, for example proposed by the NCEP/ATP III, include (1) maintenance of carbohydrate energy intake to 50%–60%; (2) protein to ~15% and (3) fat to 25%–35%, with low saturated fat intake (<7% of total energy); (4) avoidance of trans fats; (5) limited cholesterol (<300 mg/day) and refined sugar intakes (<10% of total energy) and (6) high intakes of fruits, vegetables, and whole grains [8,24,29,30]. However, some of these recommendations are under debate [31] but it is recognized that added sugar and *trans* fats, and generally high energy, refined, or processed foods have negative health effects. Furthermore, for a complex syndrome and chronic disease precursor such as MetS, the general guidelines related to reduce weight, seem to be insufficient and ask for a more scientifically based, personalized dietary approach [24]. For instance, both a high-carbohydrate and a low-carbohydrate diet, a high-protein and monounsaturated fat diet decreased all components of MetS. However each diet affected the measures of MetS differently; the low-carbohydrate diet resulted in a more successful lowering of the blood pressure (BP), while the high-carbohydrate diet led to larger decrease of LDL-cholesterol [32].

In this review, we will first describe the individual features of MetS in relation to the cerebral circulation. Most studies have examined effects of the individual MetS components on the structure and function of the vasculature rather than the impact of MetS *per se*. Moreover, only a few studies have assessed the relation of MetS and its individual components on the brain, the cerebral vasculature, and cognition. To better understand the relation between nutrition, MetS, cerebral circulation, and cognition, we focus on latest research on possible mechanisms via which nutrition may influence the individual MetS components and MetS components as a whole, and subsequent effects on the brain vasculature and circulation. We will conclude this review with the impact of nutrition on the MetS on brain functioning and cognition.

## 2. Search Strategy and Selection of the Papers

We searched both the PubMed and Web of Science databases for original and review articles published in English from 1988 to September 2015. The search strategy was based on these search terms: nutrition, obesity, dyslipidemia, hypertension, hyperglycemia, metabolic syndrome, vascular alterations, cerebrovascular circulation, cognition, and dementia. Moreover, to identify potentially relevant new papers, we filtered our total list of relevant papers by hand. Based on the title and abstract, we selected the studies. If these two components were not sufficient for selection, we purchased and evaluated the total publication.

## 3. Individual MetS Components in Relation to Cerebral Circulation

### 3.1. Abdominal Obesity

Obesity can be defined as excess body fat and an imbalance in energy intake *versus* energy output [33,34]. Body fat distribution, especially accumulation of visceral adipose tissue, is related to the diabetogenic and atherogenic abnormalities seen in MetS [35]. Moreover, MetS is relatively uncommon in the absence of excess central fat and abdominal obesity is said to be the key to MetS [36,37]. Although the body mass index (BMI) is a commonly used measure of overweight and obesity, a better marker of abdominal fat accumulation is waist circumference. Waist circumference shows a stronger correlation to visceral adiposity than BMI, but it estimates total abdominal fat, meaning both subcutaneous fat and visceral fat. Therefore, it is a surrogate marker for visceral body fat and does not correlate with blood pressure, HDL-C, and triglycerides [38].

Particularly white adipose tissue (WAT), has major endocrine, paracrine, and autocrine functions [39]. Adipocytes actively release a large number of adipokines, including leptin, adiponectin, interleukins, plasminogen activator inhibitor-1 (PAI-1), tumor necrosis factor alpha (TNF-α), adipsin (complement factor D) and growth factors [19]. Adipokines play a wide-ranging role in metabolic regulation and physiological homeostasis in both the brain and periphery [39,40]. Circulating peripheral adipokines have to cross the blood brain barrier (BBB) to interact directly with the brain [19]. Besides permeation of selective adipokines across the BBB, other interactions of adipokines with the BBB and cerebral circulation are mediated through changing endothelial function and signaling like modifying cytokine expression of brain endothelial cells or affecting vascular health inducing hypertension and thrombosis via PAI-1, by inhibiting fibrinolysis through inhibition of tissue-type plasminogen activator [41,42]. Leptin, TNF-α, and interleukin-6 (IL-6) are able to cross the BBB [19], while adiponectin affects, e.g., pro-inflammatory signals by suppressing IL-6 from endothelial cells in brain microvessels in mice [43]. It is plausible that adipokines alter membrane functions involving metabolism of lipids [44], carbohydrates, and proteins at the BBB level [42], but the direct effects of metabolic imbalance on the BBB need to be established [23].

Different metabolic profiles, accompanied by also different implications, have been found in WAT. For instance, visceral WAT secretes more pro-inflammatory adipokines than subcutaneous WAT [45]. The excessive accumulation of central body fat in obesity causes a dysregulation in the production or secretion of proinflammatory and anti-inflammatory adipokines which influences local or systemic inflammatory responses. Eventually this can lead to the initiation or progression of several obesity-linked co-morbidities including CVD, metabolic abnormalities, and neurological disorders [46]. For instance, adiponectin has important anti-atherogenic, anti-inflammatory, insulin-sensitizing, lipid-oxidation enhancing and vasodilatory properties. Reduced plasma adiponectin levels have been found in obese subjects and it has been shown that plasma adiponectin concentrations are significantly correlated with each individual component of MetS. Moreover, the multiplicity of the risk factors was increased in subjects with lower plasma adiponectin level [47]. Visceral fat is associated with carotid atherosclerosis contributing to endothelial dysfunction through the direct effect of mainly adiponectin and TNF-α, both secreted after macrophage recruitment through MCP-1. The adipokines TNF-α and interleukin 6 (IL-6) may furthermore indirectly influence inflammation processes and endothelial dysfunction [48].

Obesity promotes a state of chronic low-grade inflammation in WAT mediated by increased plasma concentrations of several adipokines, including TNF-α, IL-6, and C-reactive protein (CRP) [49,50]. Furthermore, macrophage infiltration and adipocytes differentiating into macrophages in WAT is increased, which is probably an important source of locally produced pro-inflammatory cytokines [51]. The cross-talk between the macrophages and adipocytes contributes to cytokine production and exacerbation of the metabolic activity of the WAT itself [52]. The inflammatory status evolved in response to excessive nutrient intake, is deeply involved in the development of IR and predicts the development of MetS and T2DM [49]. The resistance to insulin action promotes inflammation further via increased free fatty acids (FFA) concentrations and interference with the early on anti-inflammatory effect of insulin [52]. As a result of the inflammatory state, vascular homeostasis is altered, determined by an imbalance between the protective effect of the nitric oxide pathway and the unfavorable action of endothelin-1 produced by vascular endothelial cells [53].

Under normal conditions the endothelium actively decreases vascular tone and leucocyte adhesion, and therewith inflammatory activity in the vessel wall, limiting activation of the coagulation cascade by several pathway inhibitor interactions and regulating fibrinolysis by producing tissue plasminogen activator (tPA) and its inhibitor PAI-1. Impairment of the endothelial function, induced by components of MetS, is known to play an important role in the initiation and progression of atherosclerosis [54]. Coagulation and fibrinolytic abnormalities are often seen in obesity, suggesting that obesity induces a prothrombotic state probably as a consequence of endothelial dysfunction [55]. There are indications that visceral fat may affect the development of cerebral small vessel disease (SVD) independent of carotid atherosclerosis, possibly via inflammatory responses and IR [56]. An independent positive association was observed between increased waist circumference with Common Carotid Artery intima-media thickness, as well as with pulse wave velocity, which is a marker of elevated rigidity of the vascular wall and low distensibility [57]. Furthermore, obesity is characterized by impaired microvascular function, probably mediated by elevated FFA levels and blood glucose and an altered release of adipokines, which may contribute to the development of microangiopathy, hypertension, and IR [58].

### 3.2. Dyslipidemia

Abdominal fat accumulation is also associated with changes in lipid metabolism, including hypertriglyceridemia, reduced HDL-C levels, and increased numbers of small low density lipoprotein (LDL) particles [59]. Visceral obesity is furthermore associated with elevated FFA [60], and an excess of lipids derived from high FFA levels may lead to hypertriglyceridemia. This atherogenic dyslipidemia triad, consisting of elevated serum triglycerides, low HDL-C and an increased amount of small LDL particles, is typically part of MetS [61]. This dyslipidemia profile is also commonly observed in individuals with T2DM [62].

High serum triglyceride levels are atherogenic and a causative risk factor for coronary artery disease [61,63]. Among triglyceride-rich lipoproteins, remnant proteins and small LDL particles are generally considered as the most atherogenic agents [9]. Normally, HDL is protective against atherosclerosis by, e.g., removing excess cholesterol from peripheral tissues and by inhibiting lipoprotein oxidation [64] whereas low HDL levels as seen in MetS, seem to be independently atherogenic through multiple mechanisms [65]. Across two large clinical trials, an approximately 50% greater risk of major coronary events in subjects with MetS was found, whereby low HDL-C levels were most closely associated with this increased risk [66].

Numerous studies have revealed associations between hyperlipidemia, IR, elevated blood pressure, CVD, and coronary artery disease (CAD). The dyslipidemia profile seen in MetS is likely to interact with the renin-angiotensin-aldosterone system (RAAS), which plays a crucial role in blood pressure regulation. Potential sides for these interactions are the regulation of RAAS components in dyslipidemic conditions in cell types contributing to vascular disease and the impact of angiotensin II (ANG II) on the promotion of oxidation of LDL-C [67].

### 3.3. Hypertension

Hypertension is the third criterion regarding the presence of MetS in individuals and it is characterized by elevations in blood pressure above 140/90 mmHg in comparison with a blood pressure below 120/90 mmHg in healthy people [9,68]. Pre-hypertension, defined as blood pressure in the range of 120–139 mmHg systolic and 80–89 mmHg diastolic, increases the risk for progression to hypertension [69]. Individuals on antihypertensive medication or considered as pre-hypertensive having a blood pressure above 130/85 mmHg, might have MetS according to the NCEP/ATP III criteria [9].

Multifactorial causes appear to underlie elevated blood pressure in MetS, however three main mechanisms are likely to be involved, including increased RAAS, sympathic hyperactivation, and endothelial dysfunction, the latter being considered as the most important mechanism [70]. Hypercholesterolemia, high-fat environments, and hyperglycemia, are associated with MetS and regulate the expression of RAAS components among different cell types, including adipocytes, vascular wall cells and macrophages [67]. RAAS is expressed in adipose tissue and several studies suggest that RAAS substantially controls the synthesis of PAI-1; this adipokine is considered as the main inhibitor of the fibrinolytic system [71]. As a consequence of the excess visceral adipose tissue, increased levels of PAI-1 can affect vascular health through the inhibition of tissue-type plasminogen activator and urokinase plasminogen activator [40]. Furthermore, elevated PAI-1 is associated with atherothrombosis and it exerts a direct effect on the development of IR and T2DM [72]. Not only visceral fat is metabolically active, but also the perivascular adipose tissue (PVAT) has the ability to release a large amount of mediators contributing to the maintenance of vascular homeostasis [73]. Overactivation of the sympathic system can be caused by increased plasma levels of leptin, secondary to leptin resistance in MetS [70], or by hyperlipidemia, particularly the high circulating levels of FFA [74]. As result of an imbalance in vasodilating and vasoconstrictive factors such as nitric oxide (NO), endothelin-1 (ET-1) and urotensin-II (UT-II), endothelial dysfunction can lead to the development of hypertension via multiple mechanisms [70].

Hypertension has profound effects on the structure and function of cerebral blood vessels [75]. It promotes, for instance, the formation of atherosclerotic plaques, changing the micro turbulent flow and reducing the vascular diameter [76] which directly leads to an increased vascular resistance. Eventually, this may result in arterial occlusions and ischemic injury [77]. Hypertension also leads to vascular stiffening whereby the collagen content and rigidity of the vessel wall is increased [78]. Furthermore, hypertension induces adaptive changes in systemic and cerebral arteries, also called hypertrophic and eutrophic remodeling [75]. In hypertrophic remodeling muscle cells undergo hyperplasia or hypertrophy and grow into the lumen of the artery, resulting in an increased wall thickness and reduced outer vessel lumen [78,79]. In eutrophic remodeling smooth muscle cells undergo a rearrangement causing a reduction in outer and lumen diameter [78,79]. Normally, both adaptive responses reduce stress on the vessel wall and protect micro vessels against the negative effects of increased blood pressure. Failure of these adaptive mechanisms however, induces BBB alterations, cerebrovascular pathology, and is associated with functional and structural alterations in the neurovascular unit [75].

### 3.4. Hyperglycemia

Hyperglycemia is often preceded and accompanied by IR; a proposed secondary major underlying mechanism for MetS that is defined as a reduced sensitivity to insulin stimulation of glucose uptake and/or production [80]. Moreover, IR is associated with the development of obesity and T2DM. When excess adipose tissue is present, both the liver and muscles become overloaded with nonesterified free fatty acids (NFFAs), eventually resulting in elevated hepatic glucose output and IR in both muscle and adipose tissue [5]. IR on its turn drives the effects of MetS on the brain, likely due to the associated abnormalities in peripheral vascular reactivity and impaired endothelial function [81].

Hyperglycemia causes a large number of vascular alterations at the cellular level that are involved in, and possibly accelerate the atherosclerotic process. Three major mechanisms that promote atherosclerosis have been proposed, including non-enzymatic glycosylation of protein and lipids, oxidative stress, metabolic inflammation (metaflammation; see [12]) and protein kinase C (PKC) activity. These mechanisms encompass most of the pathological alterations observed in the vasculature [82]. For example, early glycosylation products, formed through a non-enzymatic reaction between glucose and proteins or lipoproteins in the arterial wall, may undergo a complex series of alterations that eventually lead to the formation of stable and irreversible advanced glycosylation end products (AGEs). These AGEs accumulate on the vessel wall proteins and can accelerate the atherosclerotic process via diverse receptor and non-receptor mediated mechanisms [82]. Furthermore, hyperglycemia induces a variety of biochemical changes within endothelial cells of the cerebral vasculature. Here, glucose is continuously transported from the blood by the non-insulin sensitive glucose transporter type 1 (GLUT-1). As a result, extracellular hyperglycemia induces intracellular hyperglycemia, which forms the basis of many biochemical alterations found in diabetic complications as atherosclerosis [83,84].

### 3.5. Clustering of MetS Components

Abdominal obesity, or the causes of excess unmetabolized energy in central adipose, hypertriglyceridemia, and hyperglycemia, seem to be the key drivers in the development of MetS and its consequences on cerebral circulation, accompanied by the major secondary phenomenon IR. Although several associations and interactions have been found, the most likely being a relative shortage of anti-oxidants and other cell protective micronutrients required by humans, the underlying mechanisms remain elusive. Moreover, the mechanisms by which MetS components seem to be interrelated are numerous and complex. Excess visceral body fat may induce IR, a pro-inflammatory status, and endothelial dysfunction. This might be the result of metabolic alterations, e.g., dysregulation of the production and secretion of several adipokines and elevated FFAs. Via multiple mechanisms this might lead to the development of atherogenic dyslipidemia, hyperglycemia, and hypertension, which are involved in the atherosclerotic process and can affect endothelial function. These features of MetS can induce structural and functional alterations in the vasculature, such as vascular resistance, stiffening, and remodeling, see Table 1, contributing to the development of cerebral small vessel disease and potentially affecting CBF. Studies revealed that in middle-aged subjects, MetS is adversely associated with both structure and function of large arteries, including common carotid artery intima-media thickness (IMT) and arterial stiffness [57,85]. CBF is closely related to, e.g., carotid IMT and an inverse association is found between MetS components and CBF [15]. Furthermore, MetS seems to play an important role in inducing a lowered capillary density, also known as capillary rarefaction, to such an extent as to affect cerebral auto-regulation mechanisms and cerebral arterial vasodilation reserves. These damaging effects might accelerate cerebral ischemic distress, favor the occurrence of stroke and induce cognitive impairment [86].

## 4. Relation between MetS, Cerebral Circulation, and Cognition

To maintain the structural and functional integrity of the brain, a well-regulated and continuous cerebral blood supply is crucial. For this reason it is not surprising that alterations in the cerebral vasculature, as seen in MetS, have a profound impact on cognitive function [87]. The structural and functional alterations in the blood vessels due to MetS can affect the brain by accelerating cerebral small vessel disease that may result in cerebral microbleeds, white matter lesions (WML), and brain atrophy [88]. Among MetS components, obesity and hypertriglyceridemia are associated with smaller brain volume, whereas hypertension is associated with an increased occurrence of white matter hyperintensities (WMH) and infarcts [89]. Unfortunately, the components of MetS have often been considered in an independent manner, as if the syndrome consists of a sum of different disorders with neuronal damage as the end stage where all components’ effects assemble [90]. However, this view will be reductive to give an explanation for the global metabolic effects on cognitive decline and therefore it has been suggested to consider the metabolic alterations as a continuum leading to various degrees of cognitive disorders [90,91].

A few studies assessed cognitive performance associated with MetS *per se* instead of the individual effects of the components, see Table 1. Cross-sectional studies, for instance, showed that subjects with MetS have lower mean levels in cognitive performance than control subjects on the domain of information processing speed [92,93], attention [93], and executive function [93,94]. In addition, longitudinal studies showed an association with decreased verbal fluency over 14 years [95] and a decline in global cognitive functioning over a three-year period [96]. These changes in cognition might be the result of an impaired microstructural brain tissue integrity, since microstructural brain tissue decline was found in association with MetS in middle-aged to elderly subjects. Furthermore, early confluent WMH, as detected by magnetic resonance imaging (MRI) studies, are related to vascular cognitive impairment. These microstructural changes may occur in the normal appearing brain tissue preceding macrostructural brain tissue damage [88]. For instance, the presence of cerebral WML is an important prognostic factor for the development of stroke and in the pathogenesis of cognitive deficits and dementia. Furthermore, diffuse and subtle changes in gray matter microstructure might represent an overall setup for brain damage preceding actual focal ischemic lesions, brain atrophy, and eventually cognitive impairment [97]. Mild cognitive impairment (MCI) can be considered as an intermediate stage of cognitive impairment wherein cognitive changes found in normal aging often proceed into those changes typically observed in dementia [98]. Indeed, one study described that MetS related risk for progression from MCI to dementia was increased significantly in a 3.5 year follow-up [99]. Other studies detected an association between MetS and Alzheimer’s disease (AD) [100,101], neurodegenerative diseases [102], and vascular dementia (VaD) [103,104].

**Table 1:** Overview of the effects induced by MetS on vasculature and circulation as well as the effects of Metabolic Syndrome (MetS) on cognition.

Effects of MetS on Vasculature and Circulation	Effects of MetS on Cognition
↓ Capillary density [86,105]	↓ Immediate memory function [22] ^b^
↓ Cerebral arterial vasodilation response [86]	↓ Fluid intelligence [92] ^e^
↓ Capillary recruitment [105]	↓ Global cognition [92] ^e^, [106] ^f^, [107] and in female [108] ^a^
↑ Intima media thickness [57,85] ^a^	↓ Information processing speed [93,109,110]
↑ Arterial stiffness [57,85] ^a^	↓ Executive function [109,110] ^a^, [93] and in male [94] ^a^
↓ Cerebral blood flow (CBF) [22] ^b^ and in male [15]	↓ Attention [109] ^a^ and [93]
↑ Amount of atherosclerosis [111] ^c^	↓ In recall performance [106] ^f^
↓ Vasomotor reactivity (VMR) [112] ^d^	↓ Visuospatial function [113] ^a^
↓ CBF in medial + lateral aspects of frontal & parietal lobe gray matter (GM) and lateral areas of the temporal & occipital lobe GM [22] ^b^	↓ Memory function [113] ^a^ and in male [94] ^a^

MetS is defined as the presence of ≥3 of the National Cholesterol Education Program Adult Treatment Panel III (NCEP/ATP III) criteria [8,9]. Modifications herein are: ^a^ Fasting plasma glucose ≥5.6 mmol/L (≥110 mg/dL); ^b^ BMI >25 kg/m^2^ instead of WHR; ^c^ Triglyceride levels ≥1.69 mmol/L, serum glucose ≥5.6 mmol/L (≥110 mg/dL) and HDL-C in men >1.04 mmol/L; ^d^ >80 cm waist circumference in women; ^e^ Blood pressure ≥160/90 mmHg (adjusted for an older population) and 0.247 mmol/L for fructosamine corresponds to 6.1 mmol/L for fasting plasma glucose; ^f^ No fasting blood glucose levels but insulin resistance: QUICKI of < 0.350 (1/(log (fasting insulin) + log (fasting glucose))).

The current epidemic of MetS in middle-age and the increasing prevalence of dementia with age, particularly Alzheimer’s disease, converge in findings that MetS occurring in middle-age is associated with an increased risk of cognitive decline and dementia later in life [22]. Several studies indeed indicate that MetS is a risk factor for lower cognitive function and dementia [114] and this association seems to be age-dependent [115,116,117]. It has been shown, for instance, that MetS increases the risk of cognitive decline among subjects with age up to 75 years, but with further increasing age this association starts diminishing. Moreover, among people at an age above 85 years, also known as the oldest old, this association can disappear or even reverse. Similar effects of individual MetS components, including hypertension, dyslipidemia, and obesity, are found in the oldest old [115,116]. Suggestions from other studies emerge that the critical period when cardiovascular risk factors influence cognition is during midlife [118,119]. Global and regional CBF reductions were found in subjects with MCI and AD, indicating that cerebral hypoperfusion is an underlying mechanism of neural damage and cognitive decline. In fact, CBF is compromised in MetS and both CBF and MetS are associated with lower memory performance [22]. The disturbed neurovascular unit as a consequence of MetS might cause these CBF alterations, depleting vascular reserves and disrupting the blood-brain-barrier (BBB). Moreover, the structural and functional alterations of the neurovascular unit in specific brain regions involved in memory and cognitive processes promote neuronal dysfunction and neuronal death underlying cognitive impairment [120].

The effects of MetS and IR on the brain might, next to alterations in brain perfusion, also be related to the vascular reactivity abnormalities that are associated with those conditions [121]. Glucose is the main metabolic energy source of the brain and in case the regional demand in the brain for glucose is increased, as for example, when performing a cognitive task, the main way to transport extra glucose across the blood-brain-barrier (BBB) is to relax the capillary bed, thereby exposing additional glucose transporters to the blood. Both brain perfusion and cerebral vascular reactivity play a key role in the maintenance of energy-dependent processes by generating sufficient metabolic substrates as well as clearing the “waste products” obtained from neuronal activity [122]. Subjects with MetS and/or IR are unable to maintain an optimal neuronal environment, especially in case of high metabolic demand such as during regional brain activation. This impairment, along with other factors associated with MetS such as inflammation and increased oxidative stress, can damage brain integrity [81] possibly leading to changes in cognition.

Another explanation for the affected brain functioning associated with MetS can be found in the altered production and secretion of peripheral adipokines as a consequence of excess WAT. As mentioned before, at least four adipokines, adiponectin, leptin, TNF-α, and IL-6, are able to cross the BBB and directly affect brain function [19]. Peripheral leptin enters the cerebrospinal fluid (CSF) and the central nervous system (CNS), by crossing the BBB and choroid plexus, interacting in the CNS with specific brain areas including the hypothalamus and hippocampus [123,124]. High circulating levels of leptin associated with obesity can induce leptin resistance, diminished responsiveness to leptin and decreased leptin levels in the brain. This will result eventually in an affected brain structure and impairment of memory, because leptin is involved in neurogenesis, axon growth, synaptogenesis neuroprotection, and improvement of hippocampal memory formation [19]. The adipokine TNF-α is an important mediator of inflammation seen in obesity in both the periphery and hypothalamus. Within the brain TNF-α exerts a variety of functions such as neurogenesis, synaptic transmission, and synaptic plasticity [19]. Elevated TNF-α levels are observed in several neuropathological states associated with learning and memory impairments [125]. Higher peripheral plasma IL-6 levels were associated with lower hippocampal grey matter volume in middle-aged subjects, suggesting a mediating role for peripheral IL-6 in cognitive decline [126]*.* Furthermore, elderly with MetS with high levels of serum markers of inflammation, including IL-6, showed an increased rate of cognitive impairment in comparison with those with low inflammation, implicating that inflammation at least partly modifies this increased risk [127]. Additionally, leptin, TNF-α, and IL-6 are all related to IR [51] that on its turn can contribute to the development of neurodegenerative diseases including AD [102].

## 5. Impact of Nutrition on MetS, Cerebral Circulation, and Cognition

### 5.1. Abdominal Obesity

Dietary modifications are widely known to play an important role in MetS and obesity. Weight reduction and maintenance of a lower weight had been thought to be best achieved by a combination of reduced intake of energy and increased physical activity [10]. Generally the first aim is to achieve a loss of weight of 7% to 10% from baseline total body weight during a period of 6 to 12 months. Achieving this weight loss, will generally reduce the severity of one or more MetS features regardless the type of energy restriction intervention, however weight loss is generally not maintained unless concurrent dietary quality is significantly increased [26]. For instance, both low fat, and carbohydrate restricted diets seem to be effective in reducing weight and improve the metabolic profile. However, these diets affect other metabolic markers of MetS via different mechanisms [128]. Several studies have demonstrated changes in adipokine concentrations in response to weight loss. For instance, decreased levels of leptin and IL-6 were found in obese individuals with IR [129] and an increase in adiponectin levels was detected in obese people that received gastric partition surgery [130]. Obesity is a marker of underlying inflammatory and metabolic diseases. As described earlier, adipose tissue produces several adipokines but also many pro- and anti-inflammatory cytokines like interleukin (IL)-4, IL-6, tumor necrosis factor α. The impact on obesity-based inflammation of interventions with non-whole food preparations of mono-, oligo-, or multi-item supplements such as phytoestrogens, magnesium, vitamin A and C, flavonoids, *n*-3 PUFAs and others, have been investigated, however effects of many of them are inconsistent (for more detailed information see [131]). Understandably, adherence to a traditional Mediterranean-style diet containing many of these described dietary components is associated with a diminished inflammatory state, including reductions in the adipokines IL-6 and CRP, also when adjusted for changes in body weight [132]. A recently exploding field of research is on the effect of prebiotics and probiotics (including whole food diets containing fermentable CHO and the complex fermenting microbiota) on metabolic disorders like obesity and insulin resistance and on the interplay between nutrition and microbiota. Several studies showed both beneficial and detrimental groups of microbiota on metabolic and immune profiles of the hosts [133,134].

### 5.2. Dyslipidemia

The nutrient composition of an individual’s diet has specific effects on their lipid metabolism and therewith the management of dyslipidemia in addition to those effects determined by weight gain and loss alone [24]. Glucose and lipid metabolism are strongly related, and any derangement of carbohydrate metabolism induced by a high-carbohydrate diet will also affect lipid metabolism [135]. For example, high-carbohydrate diets increase plasma triglyceride levels compared with diets consisting of higher percentages of fat in obese subjects with MetS [32]. When replacing high-carbohydrate diets for high-monounsaturated-fat diets, plasma triglyceride levels decrease [136]. Low-carbohydrate diets reduce very-low-density lipoprotein (VLDL) production and contrarily, high dietary carbohydrate leads to increased production of larger triglyceride-enriched VLDL particles, which contribute to the increased formation of small LDL particles and low HDL-C seen in MetS [135]. Low carbohydrate diets are often accompanied by a larger amount of saturated fat contributing to increased levels of triglycerides [24], while *n*-3 long chain polyunsaturated fatty acids (*n-3*-LCPUFAs) present in fatty fish and some leafy vegetables can reduce plasma triglycerides. The intake of a high-fat diet, mainly consisting of saturated fatty acids (SFAs), is associated with diminished cognitive function, impairments in prospective memory, memory speed and flexibility, and an increased vulnerability to neurological diseases [137]. However, high monounsaturated fatty acid (MUFA) and polyunsaturated fatty acid (PUFA) energy intakes [138], and higher PUFA to SFA ratios, are associated with better cognitive performance [137]. This indicates that the type and quality of fat may have a greater impact on cognition than total fat intake.

Diets consisting of carbohydrates with a low glycemic-index (GI) like low-starch and high fiber oats, seem to improve glycemic control and lipid profiles [139], while carbohydrates with a high dietary GI as in white bread, are associated with elevated triglyceride concentrations [140] and low HDL [141]. Both human studies and experimental models show that dietary fiber consumption positively affects metabolic health [142]. Dietary fiber comprises a complex group of substances that include the indigestible, non-starch polysaccharides, cellulose and hemicellulose, oligosaccharides, pectins, gums, and waxes [143]. Importantly, fiber carries and protects myriads of micronutrients until they are processed by normal gut microbes and absorbed in the colon [144]. Evidence points towards an important role for microbiota in the development of MetS. A diet rich in saturated fats allows the expansion of pathobionts, whereas a fiber-rich diet supports the microbiota to produce short-chain fatty acids (SCFA) that promote energy expenditure and protect against inflammation and IR. SCFA are end products of the intestinal microbial fermentation [145]. Complex carbohydrates, such as dietary fiber and resistant starch, form the main substrates for SCFA production in the colon. Acetate, propionate, and butyrate are the most abundant SCFA, constituting >95% of the SCFA content [146]. Transplantation of intestinal microbiota from lean, healthy donors to subjects with MetS, led to an increased insulin sensitivity [147]. SCFA seems to have a positive effect on insulin sensitivity via different mechanisms. For instance, SCFA may improve the lipid buffering capacity of adipose tissue via attenuation of intracellular lipolysis and may increase adipogenesis and regulate chronic low-grade inflammation or directly secrete adipose tissue derived proinflammatory cytokines and chemokines [146].

### 5.3. Hypertension

Several studies have demonstrated beneficial effects of diets such as the Mediterranean-style diets [148] and the Dietary Approach to Stop Hypertension (DASH) diet on blood pressure [149]. Particularly, the DASH diet with reduced calories and increased consumption of fruit, vegetables, low-fat dairy, whole grains, and diminished saturated fat, total fat and cholesterol, and Na restricted to 2400 mg, is able to lower blood pressure in individuals with MetS [150]. Furthermore, consumption of a Nordic diet, containing whole grains, rapeseed and linseed oil, berries, fruits, vegetables, fish, nuts and low-fat dairy products for 12 weeks, reduced ambulatory BP and mean arterial pressure in subjects with MetS during weight-stable condition [151]. The Mediterranean diet, the DASH diet, and the Nordic diet share many characteristics. However, the latter two diets are enriched with low-fat dairy products, which are inversely related to MetS, enlarged waist circumference, and hypertension [150]. Dairy products are a rich source of calcium and this nutrient might play a role via blood pressure reducing capacities [152]. Dairy intake may also lower the risk of MetS by maintaining vascular endothelial function by limiting postprandial hyperglycemia mediated responses that otherwise induce oxidative stress and reduce NO bioavailability [153].

Despite the difference in low-fat dairy intake, the three dietary patterns all positively affect blood pressure. This might be ascribed to the higher intake of fruits and vegetables rich in antioxidants, vitamins, and minerals, olive oil, fish, seeds, grain, nuts and rich in poly unsaturated fatty acids, and fibers [154]. Nuts are rich in monounsaturated fatty acids and polyunsaturated fatty acids, vitamins, minerals trace, soluble fiber, and polyphenols, which are neuroprotective compounds [155]. Higher adherence to the Mediterranean diet and the independent intake of olive oil or rapeseed and linseed oil, vegetables and fish, are all associated with better global cognitive function, visual memory, attention, and executive functions in elderly at high risk for MetS. In contrast, higher frequencies of consumption of red meat and full-fat dairy products are associated with worsened cognitive functioning [156]. It has been shown in the Predimed study [157] that adherence to the Mediterranean diet enriched with nuts has beneficial effects on acute myocardial infarction, stroke, or death from cardiovascular causes, and inhibits cognitive decline. It also reduces waist circumference, shifting lipoproteins to a less atherogenic pattern in subjects at high cardiovascular risk [158].

In the setting of a healthy diet, partial substitution of carbohydrates with either proteins or monounsaturated fat to 25% of energy, can further lower blood pressure [159]. In subjects with MetS, specifically a moderate and generally sustainable low-calorie diet relatively rich in protein and monounsaturated fat and poor in carbohydrate, has also been effective in lowering blood pressure [32]. Dietary omega-3 PUFA intake is inversely related to blood pressure in both hypertensive and normotensive subjects in a cross-sectional epidemiologic study [160]. Moreover, in MetS patients, supplementation with purified *n*-3 fatty acids (EPA and DHA from fish-oil), reduced systolic blood pressure [161].

A large number of studies have shown the hypertension lowering activity of nutraceuticals supporting the use of potassium, l-arginine, vitamin C, cocoa flavonoids, beetroot juice, coenzyme Q10, melatonin, probiotics, and aged garlic extract (for review see [162]). However, many intervention studies have been inconsistent or have shown damage with administering vitamins, minerals, and supplements unless there was a clear and severe deficiency.

Moreover, dietary salt and its effect on blood pressure has been studied extensively. Blood pressure reactivity to salt, known as salt sensitivity, varies considerably between individuals [163]. One study demonstrated that the blood pressure of subjects with MetS is exquisitely sensitive to dietary salt and that salt restriction was highly effective in lowering blood pressure in these subjects [164]. In addition, a population-based dietary intervention study identified a positive and significant association between MetS and salt sensitivity of blood pressure in people without diabetes, suggesting that a reduced intake of sodium can be beneficial for individuals with MetS [165].

While the effect of Na^+^ in hypertension is well-known, the role of K^+^ is still under-explored or under-reported in studies. However recently the interdependence of Na^+^ and K^+^ in the development of hypertension is being recognized and indicates the importance of simultaneous Na^+^ restriction and increased K^+^ intake as important strategies for prevention and treatment of hypertension [166]. Potassium is a very important mineral with positive effects in hypertension regulation and doubling the K^+^ intake is associated with a significant diastolic blood pressure reduction in hypertensive subjects and this effect seems larger in patients with a higher dietary Na^+^ intake [167]. A higher K^+^ is also associated with a lower incidence of cardiovascular and cerebrovascular accidents, type 2 diabetes, left ventricular hypertrophy, heart failure, and cardiac arrhythmias, independent of blood pressure reduction [168]. It has been estimated that each 1 g increase in daily K^+^ intake and each 1 g decrease in daily Na^+^ intake decreases all-cause mortality by 20% [169]. Fresh vegetables, fruit, and animal products (fish, meat) are the natural sources of K^+^, and are naturally low in Na^+^.

### 5.4. Hyperglycemia

Plasma glucose and insulin sensitivity are highly affected by diet composition. Since dietary carbohydrate represents a major precursor of plasma glucose, it is not surprising that increasing the amount of dietary carbohydrate will elevate blood glucose levels particularly in the postprandial period. Plasma glucose concentration represents an important triggering factor for insulin release, and a diet high in carbohydrates therefore, will also lead to increased insulin levels [135,170]. On the other hand, dietary carbohydrate restriction by limiting a blood glucose source, might improve glycemic control and insulin levels. The source and quality of dietary carbohydrates may also differentially optimize insulin action and thereby affect the degree of IR. Food with a high glycemic index (GI) is positively associated with IR, whereas intakes of total dietary fiber, such as from low processed whole foods: vegetables, fruit, nuts, and seeds, and invertebrate chitin/chitosan are inversely associated with IR in a cross-sectional cohort study [139]. The GI ranks carbohydrate-containing foods according to their ability to raise blood glucose concentrations postprandially compared to a reference food of either glucose or white bread [171]. It is commonly used to estimate the insulinogenic effects of foods, although the blood response is not always proportional to the insulin response. The insulin index (II) directly quantifies the insulin secretion in response to the consumption of certain foods and takes into accounts foods with a low or no carbohydrate content. Not only carbohydrates are a major stimulus for insulin secretion, but it should be noted that foods high in protein or fat, also provoke a significant insulin response [172,173]. Therefore, the II might be a suitable concept for examining insulin exposure and the development of metabolic conditions [173]. One study showed that dietary II may be relevant in relation to plasma lipids, but not for biomarkers of glycemic control [173].

Higher intake of dietary magnesium, which is a component of nuts and many whole foods, especially animal products, but also recorded in highly studied grains, is also associated with reduced risk of IR [174,175] and T2DM [176], whereas magnesium supplementation improves measure of glucose and insulin metabolism in subjects with IR [177] and T2DM [178] as well as in generally healthy adults [179]. Diets enriched in saturated fatty acids are associated with increased IR [180], whereas a high-monounsaturated-fat diet significantly improves insulin sensitivity compared to a high-saturated-fat diet [170].

Plant polyphenols may have a beneficial effect on MetS and its individual components [181]. Animal studies show that several polyphenols like resveratrol and quercetin, which are present in a variety of fruits and vegetables, affect glucose and insulin levels and lower inflammatory cytokine levels [182]. Furthermore, polyphenols may have protective effects on the pathogenic mechanisms underlying dementia (for review see [183]), like anti-inflammatory actions, enhancing cerebral blood flow, and local endothelial repair mechanisms, or via direct protective effects on neurons [184]. Unfortunately, only a few studies have considered the effects of polyphenols in humans. For example, a population based cross-sectional study showed that higher intake of flavonoids, one of the polyphenol subclasses, was associated with lower odds of enlarged waist circumference, hypertriglyceridemia, low HDL-C, hyperglycemia, hypertension, and MetS [185]. Also serum 25-OH vitamin D levels were inversely related to BMI and serum triglyceride levels [186]. Serum 25(OH) vitamin D might influence the development of adiposity, indirectly involve lipid storage via the parathyroid hormone, or affect insulin sensitivity indirectly via RAAS [187]. Observational and prospective studies have shown associations between vitamin D, specifically its 25(OH)D form, and all features of the MetS [3]. For instance, a prospective study demonstrated vitamin D deficiency in both overweight/obese and healthy people, but serum 25(OH)D levels were significantly lower in overweight/obese MetS subjects than in healthy individuals.

## 6. Conclusions

**Figure 1:**
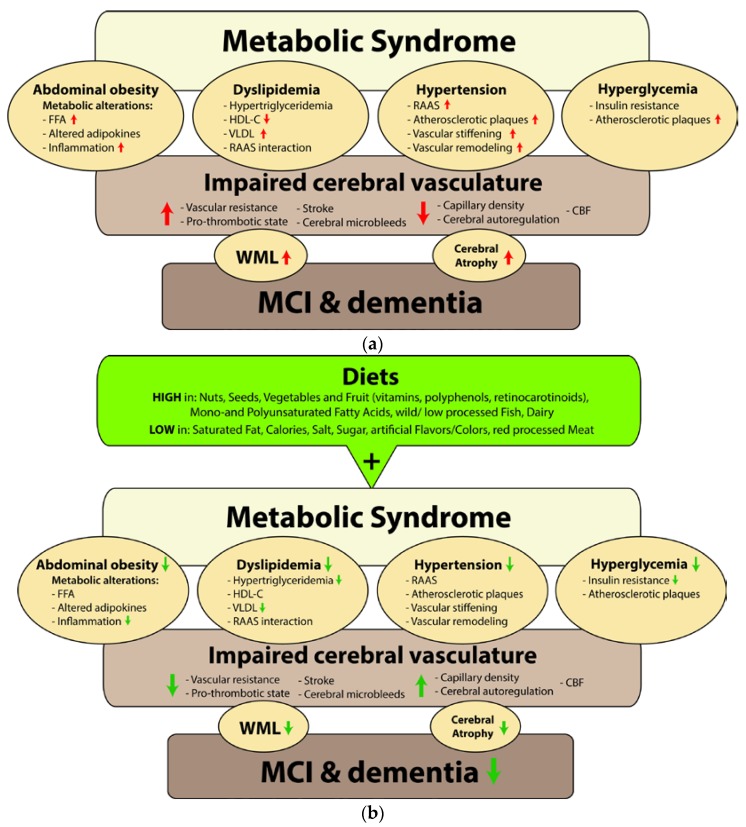
(**a**) The four components abdominal obesity, dyslipidemia, hypertension, and hyperglycemia comprising Metabolic Syndrome (MetS), contribute all to structural and functional alterations in the peripheral and brain vasculature. Associated factors highly contributing to the development of MetS are inappropriate diets physical inactivity, advancing age, and various hormones (not depicted here). Abdominal obesity is associated with metabolic alterations, including elevated FFA’s and an altered production of proinflammatory and anti-inflammatory adipokines resulting in a pro-inflammatory status. Atherogenic dyslipidemia involves hypertriglyceridemia, decreased levels of HDL-C, increased VLDL and interaction with RAAS. Increased RAAS activity is one of the mechanisms causing elevated blood pressure levels. Hypertension contributes to the atherosclerotic process and increased vascular stiffening and remodeling. Hyperglycemia is often proceeded and accompanied by IR and both influence the atherosclerotic process. Several associations and interactions are found within these processes and are also related to IR and T2DM. Alterations in the vasculature might lead to an increased vascular resistance, pro-thrombotic status, stroke, and microbleeds, while reductions are found in capillary density, cerebral autoregulation, and CBF. These features can accelerate the development of WML and cerebral atrophy, which eventually increase the risk of developing MCI and dementia. FFA = free fatty acids, HDL-C = high density lipoprotein cholesterol, VLDL = very-low-density lipoprotein, RAAS = renin-angiotensin-aldosterone-system, IR = insulin resistance, T2DM = type 2 diabetes mellitus, CBF = cerebral blood flow, WML = white matter lesions, MCI = mild cognitive impairment; (**b**) Scheme of the potential influence of dietary components on MetS and related impairment of cerebral vasculature and cognition [148,149,151,189].

The concept of MetS has been mainly associated with the peripheral systems and in this review we broadened this concept with respect to the brain, its cerebral circulation, and the impact on cognition*.* An overview is given in Figure 1a. MetS and its features not only affect the peripheral vasculature, but also induce structural and functional alterations in the cerebral vasculature, including resistance, stiffening, and remodeling. This seems to affect the brain in multiple ways. For instance, changes in the cerebral microcirculation can affect larger vessels and CBF. Changes in cerebral microcirculation also contribute to the development of cerebral small vessel disease possibly leading to white matter lesions (WML), changes in gray matter microstructure, cerebral microbleeds, and brain cell (neuronal) atrophy. The affected brain tissue integrity might be a consequence of brain perfusion alterations, cerebral autoregulation disturbances, vascular reactivity abnormalities, or an altered production and secretion of peripheral adipokines including some oligomers of adiponectin, leptin, TNF-α, and IL-6. Leptin, TNF-α, and IL-6 are all able to cross the BBB and exert a wide range of effects on brain functioning. Eventually, all these factors together may lead to an increased risk of developing MCI, dementia, and stroke.

In the coming years, the prevalence of MetS is expected to increase and affect up to 25% of the global adult population, as well drive the twin global epidemics for T2DM and CVD. Accurate and timely management of MetS is therefore crucial to the health of the world population as well as to the global economy. The focus of managing MetS should be on a healthy lifestyle with exercise [188] and consuming an overall healthy diet, since weight reduction and weight-maintenance in the long term in general will reduce the severity of one or more MetS components. The focus should therefore be on public health scale of regulating for the provision and accessibility of healthy food, and limiting possibly by taxing refined, energy dense foods. The Mediterranean, DASH, and Nordic diets, which contain overall the same ingredients and dietary composition of high mono- and polyunsaturated fats from olive/rapeseed oil, fish and nuts, low saturated fats, whole grains, many vitamins, minerals and antioxidants from fresh fruits and vegetables/legumes, have shown in many studies a positive effect on several parameters within the four criteria of MetS (see Figure 1b). Both the macro- and micronutrient composition of an individual’s diet has specific effects on obesity, dyslipidemia, hypertension, and hyperglycemia. Therefore, a more scientifically based dietary approach is required based on the individual’s MetS profile. Overall, nutrition highly influences the development of MetS and its individual components therewith increasing the risk of developing CVD, stroke, dementia, and several other pathologies.

For future research it is important to focus on the relation between MetS, cerebral vasculature, circulation, and cognition throughout life to elucidate when, and through which mechanisms cognition is affected. Furthermore, the impact of nutrition on MetS and its features should be further assessed to develop sufficient and tailored dietary guidelines. System-based approaches might be able to address the complex interaction of MetS components, how these interactions differ in disease status and vary with age, and give better insight into the underlying molecular mechanisms.
